# Characteristics of Madariaga and Venezuelan Equine Encephalitis Virus Infections, Panama

**DOI:** 10.3201/eid3014.240182

**Published:** 2024-11

**Authors:** Luis Felipe Rivera, Carlos Lezcano-Coba, Josefrancisco Galué, Xacdiel Rodriguez, Yelissa Juarez, William M. de Souza, Zeuz Capitan-Barrios, Anayansi Valderrama, Leyda Abrego, Hector Cedeño, Carmela Jackman, Jesse J. Waggoner, Patricia V. Aguilar, Hilda Guzman, Scott C. Weaver, Robert B. Tesh, Sandra López-Vèrges, Christl A. Donnelly, Cassia F. Estofolete, Mauricio L. Nogueira, Nuno R. Faria, Nikos Vasilakis, Amy Y. Vittor, Darci R. Smith, Jean-Paul Carrera

**Affiliations:** Instituto Conmemorativo Gorgas de Estudios de la Salud, Panama City, Panama (L.F. Rivera, C. Lezcano-Coba, J. Galué, X. Rodriguez, Y. Juarez, A. Valderrama, L. Abrego, S. López-Vèrges, J.-P. Carrera); Carson Centre for Health and Ecosystem Research, La Peñita, Darién, Panama (L.F. Rivera, C. Lezcano-Coba, J. Galué, X. Rodriguez, Y. Juarez, Z. Capitan-Barrios, A. Valderrama, L. Abrego, J.-P. Carrera); Facultad Salud Pública y Administración, Universidad Peruana Cayetano Heredia, Lima, Peru (C. Lezcano-Coba); University of Kentucky, College of Medicine, Lexington, Kentucky, USA (W.M. de Souza); Universidad de Panamá, Ciudad de Panamá (Z. Capitan-Barrios, L. Abrego); Ministry of Health, Panama City (H. Cedeño, C. Jackman); Emory University, Atlanta, Georgia, USA (J.J. Waggoner), The University of Texas Medical Branch, Galveston, Texas, USA (P.V. Aguilar, H. Guzman, S.C. Weaver, R.B. Tesh, M.L. Nogueira, N. Vasilakis); University of Oxford, Oxford, UK (C.A. Donnelly, J.-P. Carrera); Faculdade de Medicina de São José do Rio Preto, São Paulo, Brazil (C.F. Estofolete, M.L. Nogueira); Imperial College London, London, UK (N.R. Faria); Faculdade de Medicina da Universidade de São Paulo, São Paulo (N.R. Faria); University of Florida, Gainesville, Florida, USA (A.Y. Vittor); Naval Medical Research Command, Fort Detrick, Maryland, USA (D.R. Smith)

**Keywords:** Madariaga virus, Venezuelan equine encephalitis, viruses, alphaviruses, vector-borne infections, outbreak investigation, Panama

## Abstract

Madariaga virus (MADV) and Venezuelan equine encephalitis virus (VEEV) are emerging arboviruses affecting rural and remote areas of Latin America. However, clinical and epidemiologic reports are limited, and outbreaks are occurring at an increasing frequency. We addressed the data gap by analyzing all available clinical and epidemiologic data of MADV and VEEV infections recorded since 1961 in Panama. A total of 168 human alphavirus encephalitis cases were detected in Panama during 1961‒2023. We described the clinical signs and symptoms and epidemiologic characteristics of those cases, and also explored signs and symptoms as potential predictors of encephalitic alphavirus infection compared with those of other arbovirus infections occurring in the region. Our results highlight the challenges for the clinical diagnosis of alphavirus disease in endemic regions with overlapping circulation of multiple arboviruses.

Arthropodborne viruses (arboviruses) infect humans worldwide and cause significant illness and death. The emergence or resurgence of some arboviruses has been increasing and poses a major global health threat (*1*). US military personnel are frequently stationed in areas where arboviruses are endemic or may emerge, which could threaten military readiness.

Venezuelan equine encephalitis virus (VEEV) is widely distributed throughout the Americas; at least 14 subtypes and varieties have been described (*2*). VEEV subtypes IAB and IC can cause explosive, large-scale epizootics in horses and spillover epidemics in humans (*3*,*4*). VEEV enzootic subtypes (i.e., VEEV ID, IE) are associated with a regular incidence of human infections by spillover from enzootic cycles that involve rodents and sylvatic mosquitoes. Evidence suggests that equine-adaptive or mosquito-adaptive mutations in the VEEV enzootic subtype ID led to the emergence of epizootic and epidemic VEEV subtypes (*3*). VEEV enzootic and endemic subtype ID infection is highly prevalent in the eastern province of Darien, Panama, where human infections are sometimes fatal and seroprevalence in some villages is up to 75% of the population (*3*). Eastern equine encephalitis virus (EEEV) was reclassified as 2 different species in 2010: EEEV in North America and Madariaga virus (MADV) in Latin America (*5*). MADV was not associated with human outbreaks before 2010, when a human outbreak was reported in Darien (*6*). Both MADV and VEEV circulated simultaneously during that outbreak, and 99 acute cases and 19 hospitalizations for encephalitis were reported. Confirmed cases included 13 for MADV, 11 for VEEV, and 1 case of co-infection. A fatal MADV infection was confirmed in the same region in 2017. Modeling of 2012 and 2017 Darien Province serosurvey data suggested that alphavirus transmission is endemic in the region (*7*). Many alphavirus disease cases appear to clinically present as a self-limited febrile illness, but persistent neurologic signs and symptoms have been reported for up to 5 years after MADV and VEEV exposure (*3*).

VEEV and MADV infections are likely underdiagnosed because of limited diagnostic tools and the inability to clinically differentiate those infections from other arboviral diseases. Some estimates report that >10% of syndromically characterized dengue cases in Central and South America may be caused by VEEV (*8*). Further complicating the mischaracterization is the increasing trend in dengue incidence over the last several decades (*9*). Chikungunya virus (CHIKV) and Zika virus (ZIKV) had not previously circulated within the Western Hemisphere until the explosive emergence in 2013 (CHIKV) and 2014 (ZIKV). Both viruses became endemic in Latin America, where they now co-circulate in dengue virus (DENV)–endemic regions (*10*). The clinical presentation of those arboviral diseases can range from asymptomatic or undifferentiated mild febrile illness to severe disease (*10*).

The increasing geographic spread and disease incidence of arbovirus infections in the Americas is a major public health concern. Undifferentiated febrile illnesses remain a diagnostic and therapeutic challenge in arbovirus-prone regions because of the lack of available tools for identifying the pathogens responsible for those clinical syndromes. Shortly after disease onset, MADV and VEEV infections are often clinically indistinguishable from other arboviral syndromes, delaying prompt care for patients at risk for more serious outcomes; MADV and VEEV have been associated with severe or even fatal outcomes. Here, we describe the clinical signs, symptoms, and epidemiologic characteristics of all reported MADV and VEEV human infections occurring in Panama during 1961–2023. In addition, we explore potential symptoms as predictors of encephalitic alphavirus infection compared with those occurring from other arbovirus infections endemic to the region.

## Materials and Methods

### Alphavirus Surveillance

We heterogeneously sourced alphavirus surveillance data in Panama from samples submitted by health center clinicians upon suspecting MADV or VEEV; the national dengue surveillance system; and during outbreak response activities. Upon suspicion of MADV or VEEV (henceforth called encephalitic alphavirus infection) in Panama, health center clinicians submit blood samples to Instituto Conmemorativo de Gorgas de Estudios de la Salud (ICGES), which serves as the national reference center for infectious-disease diagnostics in Panama. Alphavirus infections are also often identified through the national dengue surveillance system or during encephalitis outbreak response activities. The national system, instituted in 1988, initially provided centralized testing of samples from suspected dengue cases submitted by clinicians during 1993–2009, but subsequently established diagnostic capacity in all local clinical units (*11*). Some alphavirus infections were identified when cases tested negative for DENV. In addition, several alphavirus outbreak investigations have been conducted since 2010 and consist of communitywide febrile surveillance and serosurveys.

### Alphavirus Outbreak Case Definition

**Figure 1:**
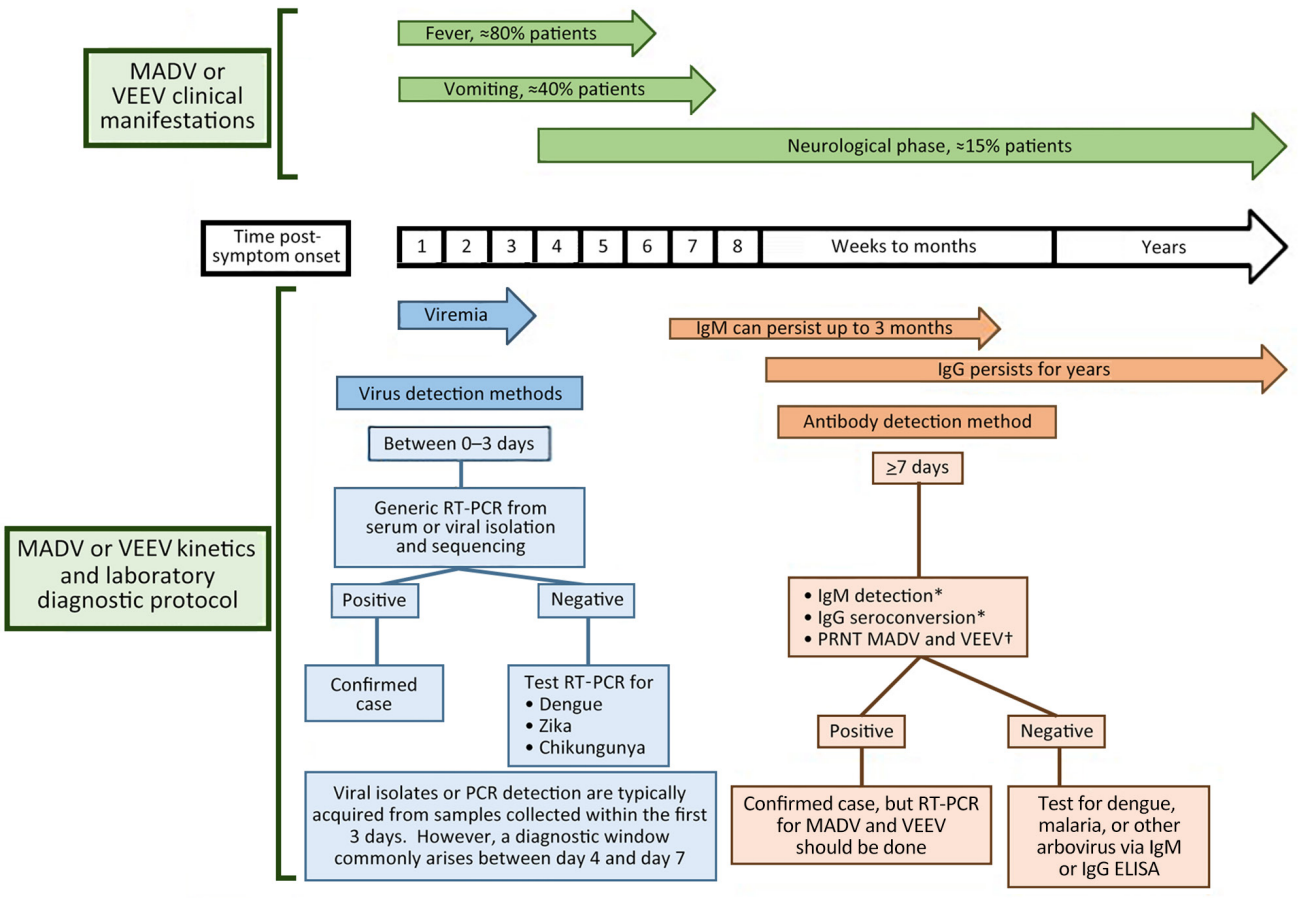
General laboratory algorithm used for diagnosis of MADV and VEEV, Panama. MADV and VEEV diagnosis made on the basis of days since symptom onset. *In paired samples showing 4-fold increase in antibody titers. †Confirmation after IgG or IgM testing. MADV, Madariaga virus; PRNT, plaque-reduction neutralization test; RT-PCR, reverse transcription PCR; VEEV, Venezuelan equine encephalitis virus.

We defined a suspected alphavirus encephalitis case as one with fever and headache, and we defined a probable case as a suspected case with neurologic manifestations (e.g., somnolence, lethargy, or seizures). We defined a confirmed case as a suspected or probable case with laboratory confirmation through viral isolation, reverse transcription PCR (RT-PCR), IgM ELISA, or IgG ELISA or plaque reduction neutralization test (PRNT) seroconversion of paired clinical samples (Figure 1; Appendix).

### Alphavirus Data Collection

We retrospectively searched and retrieved clinical and epidemiologic information of MADV and VEEV infections reported in clinical records and epidemiologic forms during 1961‒2020, data available at ICGES, and extending data published previously (*12*). Cases detected during 2021‒2023 were collected as part of the surveillance initiative undertaken by the US National Institute of Allergy and Infectious Diseases‒Centers for Research in Emerging Infectious Diseases Network initiative. The Coordinating Research on Emerging Arboviral Threats Encompassing the Neotropics in Panama and the Armed Forces Health Surveillance Division, Global Emerging Infections Surveillance Branch (ProMIS ID no. P0052_23_NM), undertake acute febrile surveillance across the country. The dataset included demographic characteristics, clinical symptoms, severity of infection, and sick contacts. We also collected geographic coordinates of alphavirus-positive households when available. We condensed duplicate or similar signs and symptoms into composite variables, which provided a better representation of the symptomatology, then used those variables to compare clinical manifestations across the main arboviral infections in Panama, including MADV, VEEV, DENV, CHIKV, and ZIKV. The Panamanian Ministry of Health (protocol no. 2077 and protocol no. 365/CBI/ICGES/2023, approved on November 30, 2023), and the Gorgas Memorial Institute institutional review board (protocol nos. 335/CBI/ICGES/21, 073/CBI/ICGES/21, and 138/CBI/ICGES/22, approved on March 19, 2021) approved the use of human data and samples from outbreaks.

### Comparison of Arboviral Symptoms

To account for low statistical power, we grouped confirmed MADV and VEEV infections into a single category. We defined encephalitic alphavirus cases as all laboratory-confirmed alphavirus infections reported in Panama during 1961–2023. We compared encephalitic alphavirus infections to DENV, ZIKV, and CHIKV. We obtained a DENV dataset from a cross-sectional study in 2009 and a ZIKV dataset from 2016. Both DENV and ZIKV datasets were provided by the São José do Rio Preto Health Service in São Paulo State, Brazil, and were published elsewhere (*13*). We obtained CHIKV data from CHIKV surveillance in the state of Amazonas, and the City of Recife, Pernambuco, Brazil, during 2015–2020 (*14*) (Appendix). 

### Statistical Methods

Initially, we included a total of 121 variables associated with participants’ symptomatology in the database; we categorized and grouped the variables by specific clinical criteria for each virus. We constructed composite symptoms based on clinical syndromic categorization by consensus of 2 independent physicians following alphavirus clinical guidelines. We further reduced symptoms using exploratory factor analysis and principal component analysis. We excluded variables with 0 variance by using a Kaiser-Meyer-Olkin threshold of 0.6 (Appendix Table 1). Ultimately, we reduced signs and symptoms to 14 variables used in the analysis.

To evaluate MADV- and VEEV-associated signs and symptoms, we conducted multivariable logistic regression analysis, controlling for age and biologic sex. We used univariate logistic regressions to evaluate the composite symptoms associated with alphavirus infection (MADV and VEEV) and those reported in DENV, ZIKV, and CHIKV infections. We selected variables using a nested log-likelihood ratio test. We excluded variables with >10% missing data from the final analysis. We expressed the associations between specific symptoms and viral infection as odds ratios; we considered p<0.05 statistically significant. We used Stata version 17 (Statacorp, https://www.stata.com) and R Studio version 2023.12.1+402.pro1 (Posit, https://www.rstudio.com) for statistical analysis.

## Results

### MADV and VEEV Epidemiology

**Figure 2:**
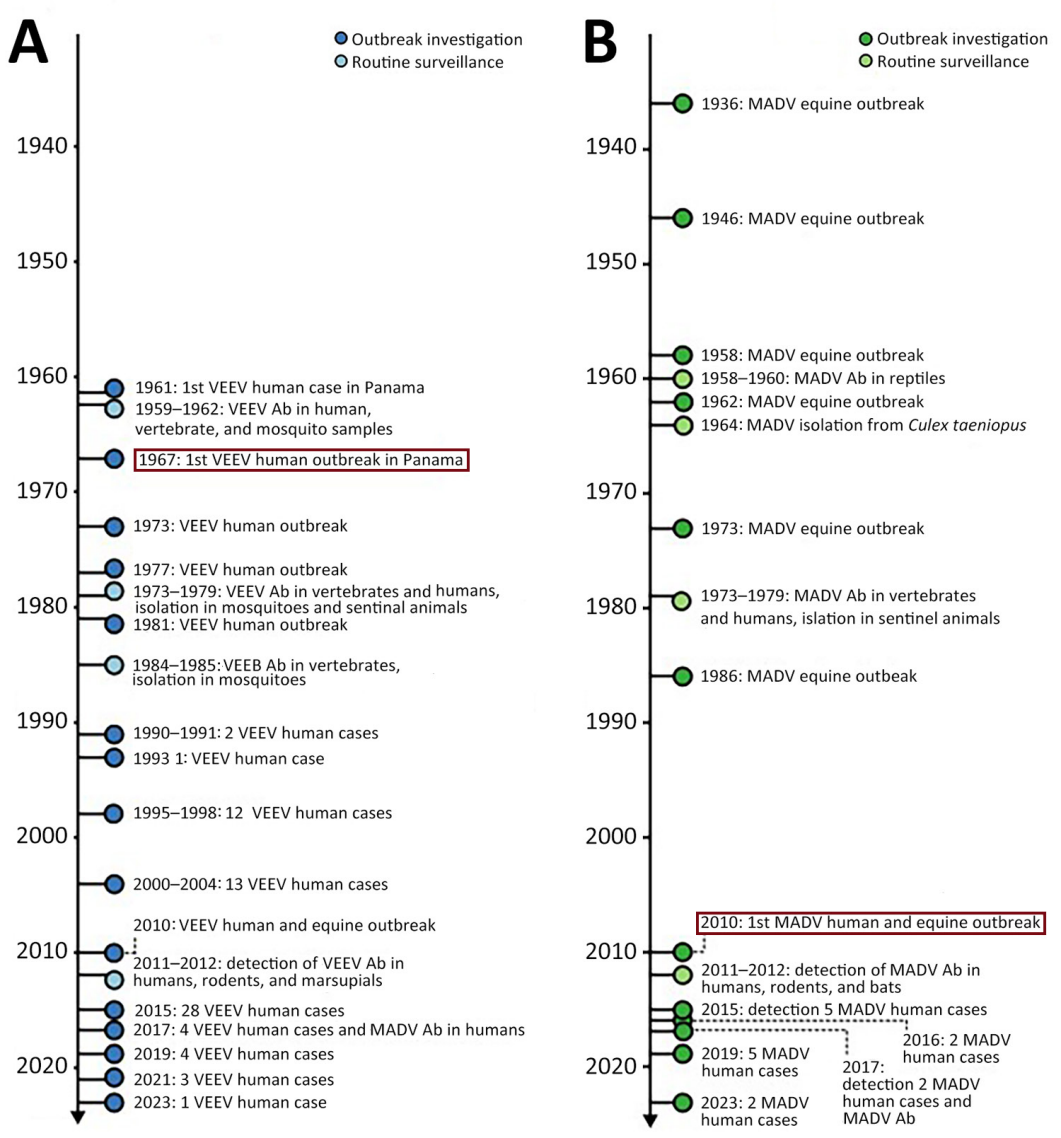
Timeline showing history of VEEV and MADV in humans animals and vectors, Panama. Timeline shows incidence during 1961‒2023. A) VEEV incidence; B) MADV incidence. Ab, antibodies; MADV, Madariaga virus; VEEV, Venezuelan equine encephalitis virus.

During 1961–2023, Panama recorded 168 laboratory-confirmed MADV or VEEV infections. For VEEV infections, 131 cases were confirmed, of which 60 (46%) were detected during outbreaks and 71 (54%) were identified through arbovirus surveillance (Table; Figure 2, panel A). For MADV infections, 37 cases were confirmed, of which 34 (92%) were identified during outbreaks and 3 (8.1%) were detected through passive arbovirus surveillance (Figure 2, panel B). Detailed clinical and epidemiologic information was accessible for 132/168 (79%) human alphavirus encephalitis infections, comprising 36 (27%) MADV infections and 96 (73%) VEEV infections. The breakdown of age distribution revealed that MADV occurred more often in children, whereas most VEEV cases occurred in adults (Table).

**Table:** Sociodemographic characteristics of cases for in a study of characteristics of Madariaga and Venezuelan equine encephalitis virus infections, Panama*

Characteristic	No. (%)
Sex	
F	163 (35.8)
M	292 (64.2)
Mean age, y (SD)	23.6 (19.7)
Age group, y	
0–5	98 (20.2)
6–20	159 (32.7)
>21	229 (47.1)
Province	
Darien	319 (71.1)
Comarca Embera	32 (7.1)
Other provinces	98 (21.8)
VEEV	
Negative	400 (80.7)
Positive	96 (19.4)
MADV	
Negative	460 (92.7)
Positive	36 (7.3)

*Samples were submitted for encephalitic alphavirus testing during 1961‒2023 (n = 496). Some variables may total <496 because of missing data. MADV, Madariaga virus; VEEV, Venezuelan equine encephalitis virus.

**Figure 3:**
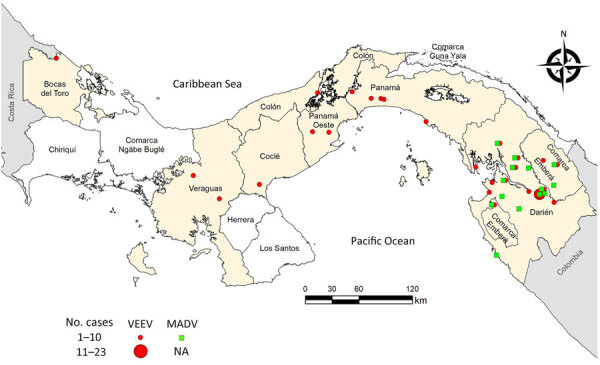
Locations of recorded cases in a study of characteristics of MADV and VEEV infections, Panama, 1961–2023. Green squares represent MADV cases and red circles VEEV cases. MADV cases were reported only in the eastern Panama region, in the province of Darien. MADV cases detected outside Darien, in Chiriquí, Comarca Näbe Bugle, and Herrera were reported in members of the border police working in the Darien Province, who at time of symptom onset were in their home region. MADV, Madariaga virus; NA, not applicable; VEEV, Venezuelan equine encephalitis virus.

All human MADV infections were reported from the Darien province. VEEV infections were reported throughout Panama, but most (63%) reports were also from Darien (Figure 3). The peak of MADV cases occurred during the 2010 outbreak in Darien, which had 13 laboratory-confirmed cases. The highest number (n = 28) of VEEV cases occurred in 2015 (Figure 2, panel A). Among the MADV case-patients, 23 were male and 13 were female. Three cases exhibited mild disease, 11 moderate, and 17 had severe clinical manifestations, resulting in a mild-to-severe ratio of 3:17. A total of 56 VEEV infections had recorded sex information; 39 case-patients were male and 17 female. Severity assessment was possible in 45 VEEV cases; 10 (22%) cases were classified as mild, 25 (56%) as moderate, and 10 (22%) as severe, resulting in a mild-to-severe ratio of 1:1. Clinical data were incomplete for 5 MADV and 51 VEEV cases. One (1/36 [2.8%]) MADV fatality and 8 (8/95 [8.4%]) VEEV fatalities were reported. 

### MADV and VEEV Laboratory Testing

We conducted a retrospective analysis to identify the diagnostic methods employed for detecting VEEV and MADV infections during 1961‒2023. MADV infections were identified nearly exclusively (n = 26 [92%]) by ELISA IgM, except a single case (8.1%) detected by RT-PCR on brain tissue after autopsy. VEEV infections were mostly identified through viral isolation (n = 67 [51%]), ELISA IgM (n = 45 [34%]), and RT-PCR (n = 19 [15%]) (Appendix Table 2).

### VEEV and MADV Clinical Presentation

**Figure 4:**
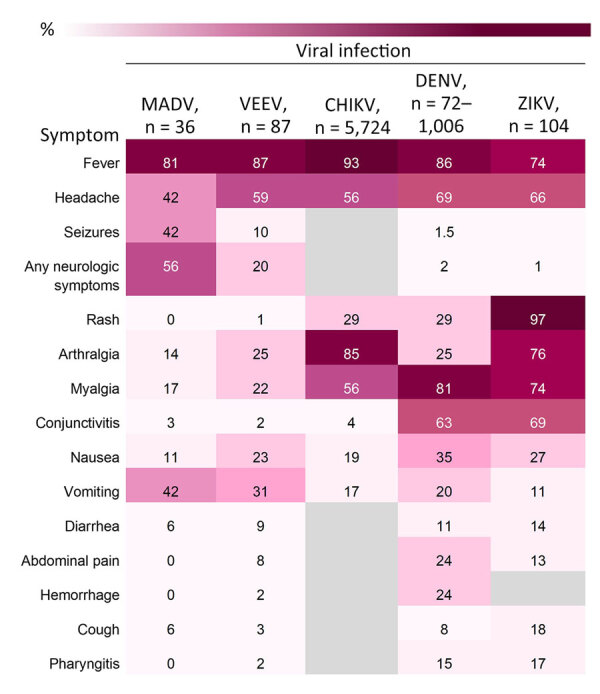
Heatmap of frequency of signs and symptoms by viral infection in South America used in study of characteristics of MADV and VEEV infections, Panama. Datasets from alphavirus cases in Panama (1961‒2023), and DENV (2009), CHIKV (2015‒2020), and ZIKV (2016) infection cases from Brazil were used to provide more complete symptom data. Gray blocks denote missing data. Neurologic symptoms included seizures, focal sensory or motor deficits, and diminished level of consciousness. CHIKV, chikungunya virus; DENV, dengue virus, MADV, Madariaga virus; VEEV, Venezuelan equine encephalitis virus; ZIKV, Zika virus.

The most frequently documented signs or symptoms of MADV and VEEV infections included fever, headache, and vomiting. Neurologic symptoms were more common in MADV infections and slightly more common among male patients. Less common signs and symptoms, including diarrhea, pharyngitis, hemorrhage, and rash, were more prevalent in VEEV infections (Figure 4).

**Figure 5:**
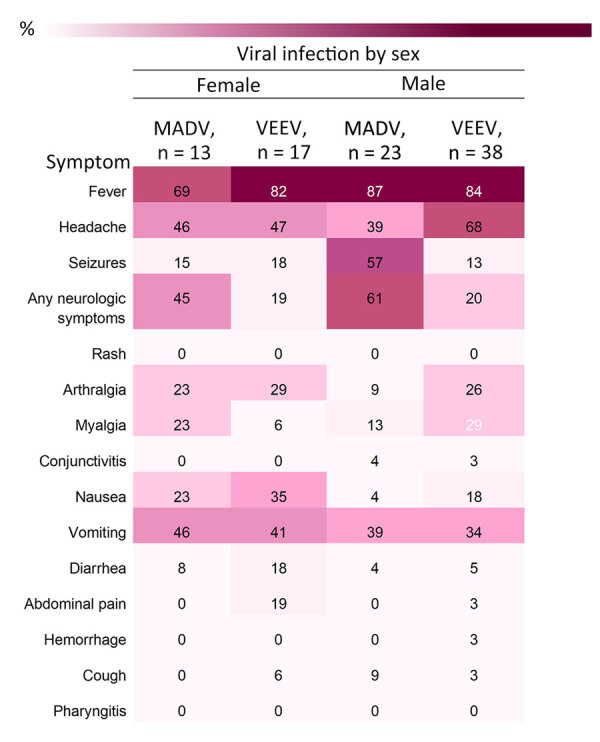
Heatmap of frequency of signs and symptoms by sex and viral infection in study of characteristics of MADV and VEEV infections, Panama. Cases reported during 1961–2023**.** Neurologic symptoms included seizures, focal sensory or motor deficits, and diminished level of consciousness. MADV, Madariaga virus; VEEV, Venezuelan equine encephalitis virus.

**Figure 6:**
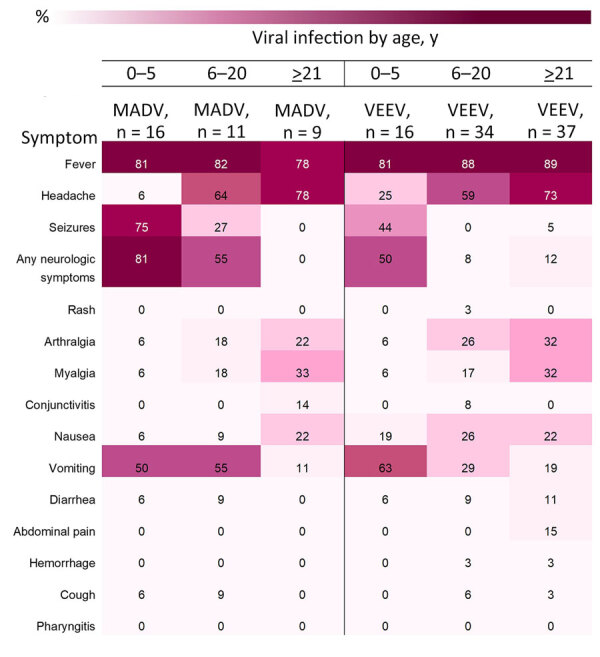
Heatmap of frequency of signs and symptoms by age and viral infection in study of characteristics of MADV and VEEV infections, Panama. Cases reported during 1961–2023**.** Neurologic symptoms included seizures, focal sensory or motor deficits, and diminished level of consciousness. MADV, Madariaga virus; VEEV, Venezuelan equine encephalitis virus.

Fever was consistently reported for both viruses, both sexes, and all age groups (Figures 5, 6). Headaches were also consistently reported in patients infected by both viruses but increased in frequency concurrent with age. Neurologic symptoms were more frequent in MADV cases in the 0–5 and 6–20 years of age groups; in contrast, neurologic symptoms were reported in the >5 years of age group of VEEV cases. The frequency of neurologic symptoms was also higher in male case-patients with MADV infections, but equally distributed among VEEV case-patients (Figure 5). Myalgia, arthralgia, and nausea were more commonly seen in VEEV case-patients, and frequency increased with age; the highest frequency was reported in the >21 years of age group (Figure 6). Abdominal pain was reported only among VEEV cases, was more common in female case-patients, and was reported exclusively in the >21 years of age group. Conjunctivitis was seen exclusively in the >21 years of age group for MADV infections. Of note, diarrhea was equally distributed among VEEV cases of both sexes until age 20; only male case-patients reported diarrhea in the >21 years of age group.

**Figure 7:**
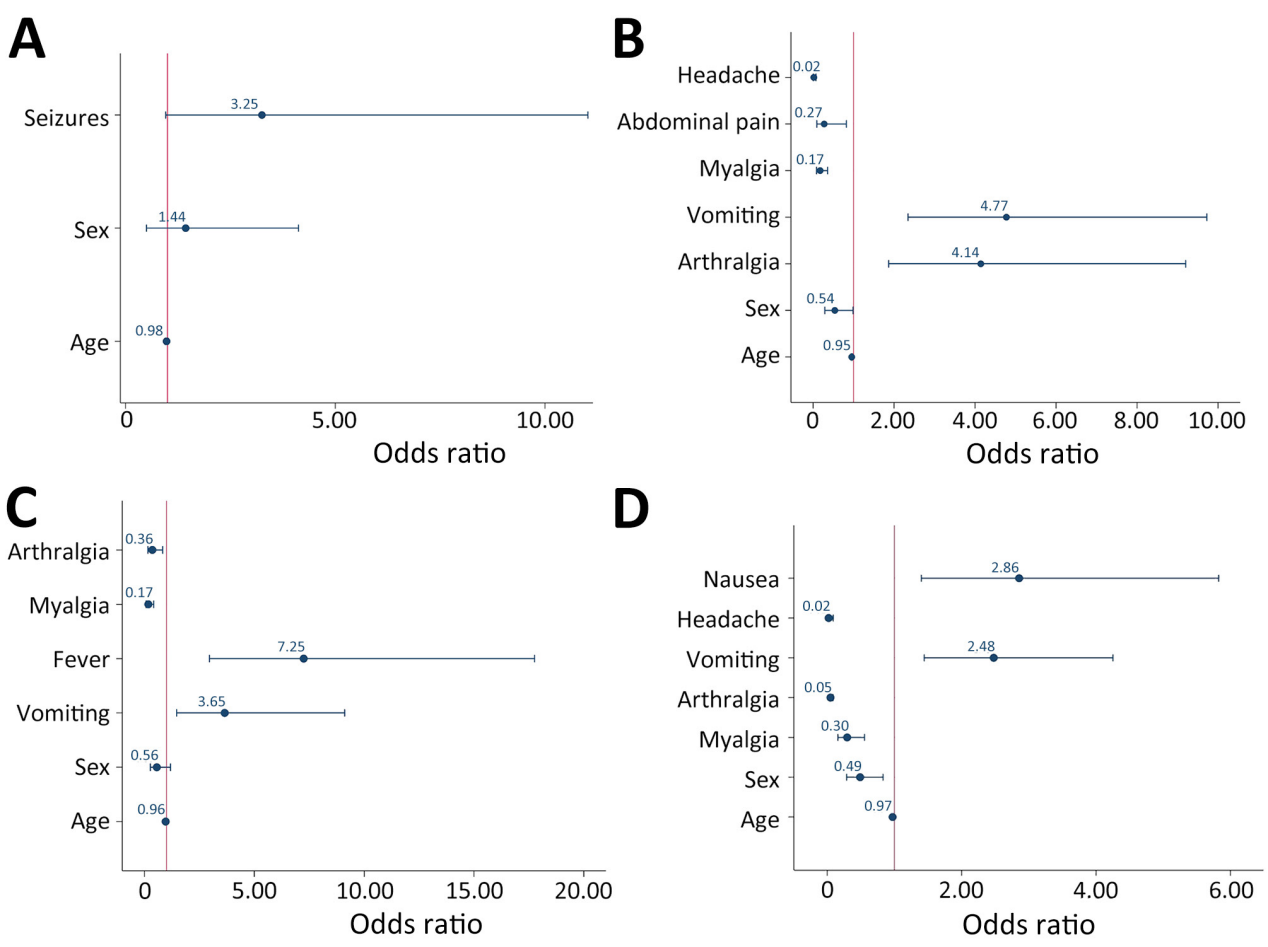
Multivariable logistic regression analysis of associated symptoms of encephalitic alphavirus infections and other arbovirus infections in study of characteristics of MADV and VEEV infections, Panama. A) MADV versus VEEV infection; B) MADV and VEEV versus DENV infection; C) MADV and VEEV versus ZIKV infection; D) MADV and VEEV versus CHIKV infection. MADV cases reported during 1961–2023**.** Dot represents odds ratio and whiskers indicate 95% CI. The red vertical line represents an odds ratio of 1, indicating that the odds of the event are the same in both groups. CHIKV, chikungunya virus; DENV, dengue virus, MADV, Madariaga virus; VEEV, Venezuelan equine encephalitis virus; ZIKV, Zika virus.

Logistic regression analysis controlling for sex and age showed that seizures and vomiting were associated with MADV infections more than VEEV infections (Appendix Table 3). At the multivariable level, after variable selection processes, only seizures remained statistically significant when comparing MADV with VEEV (Figure 7, panel A; Appendix Table 3).

### Encephalitic Alphavirus versus DENV, ZIKV, and CHIKV Infection

In multivariate analyses, dominant clinical syndromes differed by pathogen (Figure 7). Encephalitic alphavirus infections were more likely to include arthralgia and vomiting than DENV infections, and more likely to include fever and vomiting than ZIKV infections. Broadly, nausea and vomiting distinguished encephalitic alphavirus infections from CHIKV infections. We identified additional differences (Appendix Tables 4–6).

## Discussion

In this epidemiologic study, we provided a comprehensive assessment of VEEV and MADV cases in Panama. We summarized and contextualized the clinical findings of human cases of MADV and VEEV in Panama, and identified symptoms that could be considered suggestive of MADV and VEEV infection when compared with other endemic arboviral infections in the region. We have shown that MADV and VEEV cases disproportionally affected males, and that MADV occurs more often in children, whereas most VEEV cases occur in adults.

Whether sex-related or age-related susceptibility differences of VEEV and MADV are caused by the lack of preexisting immunity or different exposure risks (e.g., occupational) is unclear. VEEV has been present in Panama since the mid-20th century, when the virus was isolated from a fatal human case in 1961 (*15*). The first recorded human outbreak of VEEV in Panama occurred in 1967 in US soldiers training on the western shores of Gatun Lake (*16*). Since then, VEEV outbreaks have been periodically reported in humans. Although equine cases of MADV have been documented in Panama since 1936 (*17*), instances of human cases were infrequent before 2010, despite active human surveillance during outbreaks and widespread mosquito isolations (*8*,*18*,*19*). A 2012 study on MADV and VEEV seropositivity in humans demonstrated an increasing prevalence of antibodies for VEEV with age, demonstrating that the virus is endemic in the region (*20*). The same trend was not observed for MADV, which suggested that the virus recently emerged in humans during the 2010 outbreak. MADV may have gained human virulence since 2010 (*6*), which may explain why we continue to see human cases. Children may be more susceptible to MADV because of lack of preexisting immunity or an immature immune system. The primary risk factors for human exposure to both viruses were found to be farming and fishing (*20*); spending more time outside performing those activities may put boys and men at an increased risk for exposure to infected mosquitoes. Our results highlight the need for continued surveillance for VEEV and MADV to better understand the 2 viruses and the differences between VEEV and MADV infection.

Darien Province in Panama is a hotspot for VEEV and MADV activity, especially for more recent outbreaks. All MADV human infections have occurred in that region, whereas VEEV infections have occurred throughout Panama. Darien is a remote region in eastern Panama near the Colombia border that is inhabited primarily by Indigenous communities. The region contains swamps and forest habitats that can support the enzootic transmission cycle of VEEV and MADV, which involves rodents and mosquitoes. Both viruses have the same mosquito vectors within the subgenus *Culex* (*Melanoconion*), and potentially the same rodent reservoir (*21*). The Darien Province also has a high number of refugee and migrant crossings; the United Nations reported >500,000 crossings in 2023 (*21*). Human migration through the region could result in more cases and potential spread to other regions; the MADV cases detected outside Darien were in members of the border police working in Darien Province whose symptoms did not develop until they returned to their home regions. Although our study reports 168 confirmed human cases of encephalitic alphavirus infection in Panama, the true burden of disease is likely underestimated, which is highlighted by the recent finding that 11.9% of dengue-like disease patients had VEEV infections (*22*).

The first limitation of our study is that the tests used for regular alphavirus diagnostics were in-house tests; an RT-PCR was recently developed (*22*). Alphavirus infections in this cohort were diagnosed with a variety of tests over time as more robust methodologies were adopted. The diagnostic test performance of legacy tests performed before 2022 is not known, and misclassification bias might exist among the relevant cases. Clinical information on alphavirus infections was documented using forms that might not capture detailed clinical and laboratory parameters for both VEEV and MADV infections, and clinical data entry was incomplete. Second, encephalitic alphavirus cases often occur in rural or remote areas with limited healthcare systems and resources, which could mean the number of cases is underestimated. Third, the limited sample size could affect statistical power and conclusions, particularly for less frequent symptoms. Fourth, the clinical outcome could be virus strain‒dependent and thus vary geographically. Finally, we compared symptoms of encephalitic alphavirus infection with those of DENV, ZIKV, and CHIKV infections from cohorts in Brazil; although the genetic background and social conditions in Panama may differ from those in Brazil, symptoms of DENV, ZIKV, and CHIKV infections appear to be similar across different populations (*23*–*26*).

In summary, outbreaks of MADV and VEEV are expected to continue, highlighting the need for continued surveillance efforts in Panama and other parts of Central and South America. Our findings could serve as a valuable tool for clinical and epidemiologic decision making in regions characterized by endemic arboviral circulation and limited laboratory capacity.

This article was preprinted at https://doi.org/10.1101/2024.02.02.24302220.

Appendix Additional information about Madariaga virus and Venezuelan equine encephalitis virus infections, Panama.
