# Supplementary material for: Characteristics of Madariaga and Venezuelan Equine Encephalitis Virus Infections, Panama
Source: Emerg Infect Dis. 2024 Nov;30(Suppl 2):S94–S104. doi: 10.3201/eid3014.240182 (PMC11559567; doi:10.3201/eid3014.240182)
Supplement: Appendix — Additional information about Madariaga virus and Venezuelan equine encephalitis virus infections, Panama. [file 24-0182-Techapp-s1.pdf]

Article DOI: http://doi.org/10.3201/eid3014.240182

EID cannot ensure accessibility for supplementary materials supplied by authors. Readers who have difficulty accessing supplementary content should contact the authors for assistance.

Characteristics of Madariaga and Venezuelan Equine Encephalitis Virus Infections, Panama

Appendix

Supplementary Laboratory Procedures

Alphavirus Diagnostic

Viral Isolation and Molecular Testing

Direct viral detection was addressed using serum or cerebrospinal fluid (CSF) obtained from patients during the early acute stage of the disease (duration of symptoms, ≤3 days), was used for viral RNA extraction using QIAmp RNA viral extraction kit (QIAGEN, Valencia, CA), and attempt viral isolation in Vero cells supplemented with 2% fetal bovine serum and 1% penicillin/streptomycin. Cells were observed daily for evidence of cytopathic effects (CPE) and samples were passed twice for CPE confirmation. Brain tissues from fatal cases were used to prepare a 10% brain suspension using MEM and 10% FBS and used for viral RNA extraction and attempt viral isolation as described before. Viral RNA from serum, cell supernatant and brain suspension were tested using an alphavirus universal reverse transcription-polymerase chain reaction (RT-PCR) assay or specific MADV/VEEV Real Time RT-PCR as described previously (*1*).

Serology

Acute serum or cerebrospinal fluid (CSF) samples collected during the middle acute phase (duration of symptoms, ≤7) was tested for MADV or VEE virus IgM and IgG antibodies for paired samples, using an enzyme-linked immunosorbent assay as described previously (*2*,*3*). In summary, ELISA antigens were prepared using the TC-83 VEEV and EEEV (prepared by Dr. Robert Shope at the Yale Arbovirus Research Unit in August 1989) strain-infected mouse brain

using the sucrose-acetone method. ELISA positive samples by means of IgM or IgG were confirmed using a plaque reduction neutralization test (PRNT), with 80% of reduction as cutoff value. Strains VEEV TC-83 and MADV GML-267113 were used for PRNT confirmation.

Dengue, Zika, and Chikungunya Virus Infections

Patients with suspected DENV infection were recruited at the Hospital of the Faculdade de Medicina de São José do Rio Preto, in São Paulo. After initial care was provided, the suspected cases were reported in SINAN (Brazilian notification system). Clinical information of DENV infections was obtained from computerized medical records and disease notification forms according to the WHO 2009 guidelines and the Brazilian Ministry of Health. Blood samples were collected and subjected to relevant diagnostic assays and stored at −80°C until they were repurposed for additional testing relevant to this study. Dengue was classified as: (i) dengue without warning signs (DwWS), (ii) dengue with warning signs (DWS), or (iii) severe dengue disease (SDD).

References

1. Carrera JP, Araúz D, Rojas A, Cardozo F, Stittleburg V, Morales Claro I, et al. Real-time RT-PCR for Venezuelan equine encephalitis complex, Madariaga, and Eastern equine encephalitis viruses: application in human and mosquito public health surveillance in Panama. J Clin Microbiol. 2023;61:e0015223. PubMed https://doi.org/10.1128/jcm.00152-23

2. Carrera JP, Cucunubá ZM, Neira K, Lambert B, Pittí Y, Liscano J, et al. Endemic and epidemic human alphavirus infections in eastern Panama: an analysis of population-based cross-sectional surveys. Am J Trop Med Hyg. 2020;103:2429–37. PubMed https://doi.org/10.4269/ajtmh.20-0408

3. Vittor AY, Armien B, Gonzalez P, Carrera JP, Dominguez C, Valderrama A, et al. Epidemiology of emergent Madariaga encephalitis in a region with endemic Venezuelan equine encephalitis: initial host studies and human cross-sectional study in Darien, Panama. PLoS Negl Trop Dis. 2016;10:e0004554. PubMed https://doi.org/10.1371/journal.pntd.0004554

Appendix Table 1. Kaiser-Meyer-Olkin measure of sampling adequacy for the selected symptom variables, Principal Component Analysis (PCA)

Variable	Kaiser-Meyer-Olkin
Abdominal pain	0.5619
Fever	0.6824
Respiratory symptoms	0.6811
Mucosal bleeding	0.5275
Seizures	0.6531
Pharyngitis	0.6648
Headache	0.7039
Arthralgias	0.6224
Nausea	0.6635
Myalgias	0.6939
Rash	0.7134
Conjunctivitis	0.6632
Vomiting	0.6008
Diarrhea	0.7039
Overall	0.6554

Appendix Table 2. Distribution of MADV and VEEV cases by year, diagnostic method, and detection type

Year	MADV positive cases	MADV diagnostic methods	MADV detection method	VEEV positive cases	VEEV diagnostic methods	VEEV detection method
1961	0	NA	NA	1	Viral isolation	Arbovirus surveillance
1962	0	NA	NA	3	Viral isolation	Arbovirus surveillance
1964	0	NA	NA	1	Viral isolation	Arbovirus surveillance
1967	0	NA	NA	7	Viral isolation	Arbovirus surveillance
1973	0	NA	NA	10	Viral isolation	Arbovirus surveillance
1977	0	NA	NA	9	Viral isolation	Arbovirus surveillance
1981	0	NA	NA	1	Viral isolation	Arbovirus surveillance
		NA	NA	5	ELISA IgM	Arbovirus surveillance
1990	0	NA	NA	1	Viral isolation	Arbovirus surveillance
1991	0	NA	NA	1	Viral isolation	Arbovirus surveillance
1993	0	NA	NA	1	Viral isolation	Arbovirus surveillance
1995	0	NA	NA	2	Viral isolation	Arbovirus surveillance
1996	0	NA	NA	1	Viral isolation	Arbovirus surveillance
1997	0	NA	NA	2	Viral isolation	Arbovirus surveillance
1998	0	NA	NA	7	Viral isolation	Arbovirus surveillance
2000	0	NA	NA	1	Viral isolation	Arbovirus surveillance
2001	0	NA	NA	6	Viral isolation	Arbovirus surveillance
2002	0	NA	NA	1	Viral isolation	Arbovirus surveillance
2003	0	NA	NA	5	Viral isolation	Arbovirus surveillance
2004	0	NA	NA	3	Viral isolation	Arbovirus surveillance
2010	14	ELISA IgM	Outbreak	8	ELISA IgM	Outbreak
				1	RT-PCR	Outbreak
				3	Viral isolation	Outbreak
2015	5	ELISA IgM	Outbreak	13	RT-PCR	Outbreak
				15	ELISA IgM	Outbreak
2016	3	ELISA IgM	Outbreak			
2017	1	RT-PCR	Arbovirus surveillance	10	ELISA IgM	Outbreak
	6	ELISA IgM	Outbreak			
2019	6	ELISA IgM	Outbreak	7	ELISA IgM	Outbreak
				1	Viral isolation	Outbreak
2021				3	RT-PCR	Arbovirus surveillance
2023	2	RT-PCR	Arbovirus surveillance	2	RT-PCR	Outbreak

*MADV, Madariaga virus; NA, not applicable; RT-PCR, reverse transcription PCR; VEEV, Venezuelan equine encephalitis virus.

Appendix Table 3. Symptoms associated with MADV cases compared to VEEV controls (n = 496)*

	Univariate analysis			Multiple regression, nested model†		
Characteristics	OR	95% CI	p value	OR	95% CI	p value
Abdominal pain						
No	Referent			Referent		
Yes	0.78	0.08–7.32	0.832			
Fever						
No	Referent			Referent		
Yes	0.72	0.23–2.29	0.582			
Respiratory symptoms‡						
No	Referent			Referent		
Yes	3.12	0.59–16.39	0.180			
Mucosal bleeding‡						
No	Referent			Referent		
Yes	Empty					
Seizures						
No	Referent			Referent		
Yes	7.49	2.75–20.46	**<0.001**	3.25	0.96–11.02	0.058
Pharyngitis‡						
No	Referent			Referent		
Yes	Empty					
Headache						
No	Referent			Referent		
Yes	0.31	0.07–1.40	0.127			
Arthralgias						
No	Referent			Referent		
Yes	0.65	0.19–1.98	0.415			
Nausea						
No	Referent			Referent		
Yes	0.66	0.20–2.13	0.485			
Myalgias						
No	Referent			Referent		
Yes	0.92	0.31–2.76	0.88			
Rash**						
No	Referent			Referent		
Yes	Empty					
Conjunctivitis‡						
No	Referent			Referent		
Yes	3.25	0.19–53.66	0.410			
Vomiting						
No	Referent			Referent		
Yes	2.37	1.01–5.59	**0.049**			
Diarrhea						
No	Referent			Referent		
Yes	0.78	0.16–3.89	0.760			

*Some variables may total less than 9,644 due to missing data. Bold text indicates statistical significance. OR, odds ratio.
†Adjusted by age and sex.
‡Variables excluded from the multivariable model due to missing values.

Appendix Table 4. Symptoms associated with alphavirus cases with DENV controls (n = 9,644)*

	Univariate analysis			Multiple regression, nested model†		
Characteristics	OR	95% CI	p value	OR	95% CI	p value
Abdominal pain						
No	Referent			Referent		
Yes	0.19	0.07–0.48	**<0.001**	0.27	0.09–0.82	**0.021**
Fever						
No	Referent			Referent		
Yes	1.08	0.63–1.85	0.768			
Respiratory symptoms						
No	Referent			Referent		
Yes	5.08	1.89–13.65	**0.001**			
Mucosal bleeding						
No	Referent			Referent		
Yes	0.20	0.04–0.80	**0.024**			
Seizures‡						
No	Referent			Referent		
Yes	43.92	17.40–110.85	**<0.001**			
Pharyngitis‡						
No	Referent			Referent		

	Univariate analysis			Multiple regression, nested model†		
Characteristics	OR	95% CI	p value	OR	95% CI	p value
Yes	1.65	0.36–7.47	0.514			
Headache						
No	Referent			Referent		
Yes	0.12	0.07–0.21	**<0.001**	0.01	0.01–0.07	**<0.001**
Arthralgias						
No	Referent			Referent		
Yes	0.81	0.50–1.31	0.399	4.14	1.86–9.20	**<0.001**
Nausea						
No	Referent			Referent		
Yes	0.5	0.30–0.81	**0.005**			
Myalgias						
No	Referent			Referent		
Yes	0.09	0.05–0.15	**<0.001**	0.17	0.08–0.36	**<0.001**
Rash‡						
No	Referent			Referent		
Yes	empty					
Conjunctivitis‡						
No	Referent			Referent		
Yes	0.42	0.10–1.78	0.245			
Vomiting						
No	Referent			Referent		
Yes	2.17	1.44–3.27	**<0.001**	4.77	2.34–9.72	**<0.001**
Diarrhea‡						
No	Referent			Referent		
Yes	10.64	4.33–26.13	**<0.001**			

*Some variables may total less than 9,644 due to missing data. Bold text indicates statistical significance. OR, odds ratio.
†Adjusted by age and sex.
‡Variables excluded from the multivariable model due to missing values.

Appendix Table 5. Symptoms associated with alphavirus cases with ZIKV controls (n = 9,644)*

	Univariate analysis			Multiple regression, nested model†		
Characteristics	OR	95% CI	p value	OR	95% CI	p value
Abdominal pain						
No	Referent			Referent		
Yes	0.43	0.15–1.25	0.124			
Fever						
No	Referent			Referent		
Yes	3.70	2.00–6.83	**<0.001**	7.25	2.96–17.76	**<0.001**
Respiratory symptoms						
No	Referent			Referent		
Yes	0.32	0.12–0.84	**0.021**			
Mucosal bleeding						
No	Referent			Referent		
Yes	2.58	0.23–28.78	0.442			
Seizures‡						
No	Referent			Referent		
Yes	31.78	4.21–239.63	**0.001**			
Pharyngitis‡						
No	Referent			Referent		
Yes	0.12	0.02–0.51	**0.005**			
Headache						
No	Referent			Referent		
Yes	0.17	0.09–0.31	**<0.001**			
Arthralgias						
No	Referent			Referent		
Yes	0.17	0.09–0.29	**<0.001**	0.36	0.15–0.82	**0.016**
Nausea						
No	Referent			Referent		
Yes	0.76	0.41–1.42	0.395			
Myalgias						
No	Referent			Referent		
Yes	0.17	0.09–0.31	**<0.001**	0.17	0.07–0.42	**<0.001**

Characteristics	Univariate analysis OR	95% CI	p value	Multiple regression, nested model† OR	95% CI	p value
Rash‡						
No	Referent			Referent		
Yes	empty					
Conjunctivitis‡						
No	Referent			Referent		
Yes	0.02	0.01–0.07	**<0.001**			
Vomiting						
No	Referent			Referent		
Yes	4.79	2.41–9.51	**<0.001**	3.65	1.46–9.12	**0.006**
Diarrhea						
No	Referent			Referent		
Yes	0.72	0.31–1.65	0.439			

*Some variables may total less than 9,644 due to missing data. Bold text indicates statistical significance. OR, odds ratio.
†Adjusted by age and sex.
‡Variables excluded from the multivariable model due to missing values.

Appendix Table 6. Symptoms associated with alphavirus cases compared to CHIKV controls (n = 9,644)* †

Characteristics	Bivariate analysis OR	95% CI	p value	Multiple regression, nested model† OR	95% CI	p value
Abdominal pain‡						
No	Referent			Referent		
Yes	Empty					
Fever						
No	Referent			Referent		
Yes	0.48	0.28–0.82	**0.007**			
Respiratory symptoms‡						
No	Referent			Referent		
Yes	107.68	26.58–436.25	**<0.001**			
Mucosal bleeding‡						
No	Referent			Referent		
Yes	Empty					
Seizures‡						
No	Referent			Referent		
Yes	Empty					
Pharyngitis‡						
No	Referent			Referent		
Yes	96.86	8.72–1,075.51	**<0.001**			
Headache						
No	Referent			Referent		
Yes	0.14	0.08–0.22	**<0.001**	0.02	0.01–0.09	**<0.001**
Arthralgias						
No	Referent			Referent		
Yes	0.04	0.02–0.06	**<0.001**	0.05	0.03–0.09	**<0.001**
Nausea						
No	Referent			Referent		
Yes	0.91	0.57–1.47	0.703	2.86	1.40–5.82	**0.004**
Myalgias						
No	Referent			Referent		
Yes	0.15	0.09–0.24	**<0.001**	0.29	0.16–0.56	**<0.001**
Rash‡						
No	Referent			Referent		
Yes	Empty					
Conjunctivitis						
No	Referent			Referent		
Yes	0.42	0.10–1.70	0.225			
Vomiting						
No	Referent			Referent		
Yes	2.28	1.55–3.36	**<0.001**	2.48	1.44–4.25	**0.001**
Diarrhea‡						
No	Referent			Referent		
Yes	259.55	56.22–1,198.35	**<0.001**			

*Some variables may total less than 9,644 due to missing data. Bold text indicates statistical significance. OR, odds ratio.
†Adjusted by age and sex.
‡Variables excluded from the multivariable model due to missing values.

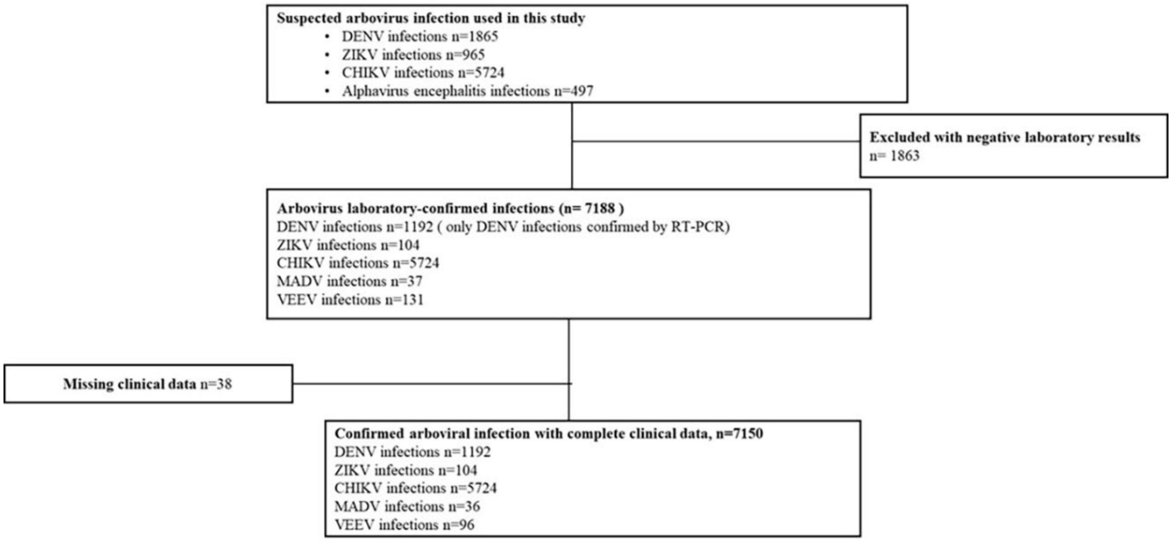

Appendix Figure. Flowchart of alphavirus and endemic arbovirus infections included in this study.
